# All-Nanochitin-Derived, Super-Compressible, Elastic, and Robust Carbon Honeycombs and Their Pressure-Sensing Properties over an Ultrawide Temperature Range

**DOI:** 10.1021/acsami.3c08587

**Published:** 2023-08-23

**Authors:** Xiang Li, Luting Zhu, Takaaki Kasuga, Masaya Nogi, Hirotaka Koga

**Affiliations:** SANKEN (The Institute of Scientific and Industrial Research), Osaka University, 8-1 Mihogaoka, Ibaraki, Osaka 567-0047, Japan

**Keywords:** nanochitin, carbon honeycomb, anisotropic porous structures, elastic carbon, ultrawide-temperature-applicable pressure sensing

## Abstract

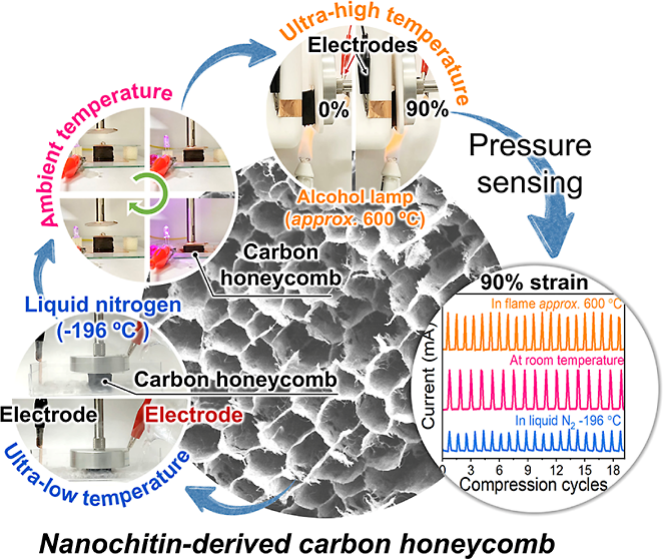

Elastic carbon aerogels show great potential for various applications but are often hindered by structure-derived fatigue failure, weak elasticity with low compressibility, and low stress and height retention. Herein, we demonstrate a super-elastic and fatigue-resistant nanochitin-derived carbon honeycomb with honeycomb-like anisotropic microstructures and carbon-based molecular structures, which was tailored by optimizing the nanochitin concentrations and carbonization temperatures. The carbon honeycomb fabricated at a nanochitin concentration of 1.0 wt % and a carbonization temperature of 900 °C demonstrated anisotropic honeycomb channels, nanofibrous network channel walls with few cracks, and weak interactions between the carbonized nanochitin, which afforded high compressibility with up to 90% strain and complete recovery. In particular, the carbon honeycomb provided good fatigue resistance with high stress and height retentions of 87 and 94%, respectively, after more than 10,000 compression cycles at 90% strain. Moreover, the tailored anisotropic honeycomb channels and molecular structures endowed the carbon honeycomb with elasticity even under severe conditions, such as exposure to flame (approximately 1000 °C) and liquid nitrogen (approximately −196 °C). Owing to these properties, the nanochitin-derived carbon honeycomb could act as a high-sensitivity pressure sensor for a wide working pressure range of 0–185.5 kPa and ultrawide temperature range of −196–600 °C. This study can provide a promising route to develop all-biomass-derived, super-elastic, and fatigue-resistant carbon materials for pressure sensing under harsh conditions and for versatile electronic applications.

## 1 Introduction

Elastic carbon aerogels are considered ideal candidates for applications such as triboelectric nanogenerators,^1^ flexible energy storage,^2,3^ and pressure sensors^4^ owing to their light weight, large specific surface area, and high electrical conductivity. Furthermore, the high thermal stability of elastic carbon aerogels can provide wide-temperature-range applicability, which is beneficial for use in harsh environments such as outer space. Compressibility, elasticity, and fatigue resistance are the three key factors that determine the performance and applications of elastic carbon aerogels.^5,6^ Owing to the highly porous structure of carbon aerogels, their high compressibility has been previously demonstrated.^7^ However, recovery to their original height and stress upon cyclic compression has been rarely achieved owing to the brittleness of random carbon networks.^8^ Therefore, achieving an elastic carbon aerogel with super-elasticity and fatigue resistance upon large cyclic compression strain is highly demanded for broadening the practical applications, which include elastic cushioning systems for vehicles and shoes to minimize mechanical shock and wide-range pressure sensors for wearable electronics.

Tailoring of the carbon precursor, carbonization conditions, and the microstructure has been exploited to fabricate elastic carbon aerogels. Fossil-derived one-dimensional, two-dimensional, and related carbon materials such as carbon nanotubes (CNTs),^9^ graphene,^7^ and CNT/graphene composite aerogels^5^ notably comprised continuous and crosslinked stacked networks as soft and flexible cell walls, preventing irreversible buckling and/or fracture upon cyclic compression. CNTs and graphene have been composited with elastic matrices such as polyurethane sponges and microcellular poly(ether-block-amide) beads by dip coating and physical microcellular foaming.^10,11^ From the perspective of sustainable development, these fossil-derived carbons have been actively composited with biomass or carbonized biomass materials to fabricate elastic carbon aerogels.^12,13^ Ideally, the complete replacement of the fossil-derived carbons with thermally or hydrothermally carbonized biomass (polysaccharides,^14^ lignin,^15^ protein,^16^ and bamboo^17,18^) offers a remarkable strategy for fabricating sustainable carbon aerogels without using non-renewable resources. Nonetheless, carbonization causes severe weight loss and volume shrinkage of the biomass-derived precursors, resulting in lower elasticity and fatigue resistance of the carbonized biomass aerogels compared to fossil-derived carbon aerogels.^19^ To address this problem, Peng et al. developed a cellulose nanofiber-/lignin-derived composite carbon aerogel;^15^ the lignin acted as a “glue” that prevented the high volume and shape shrinkage of the cellulose nanofibers, thus realizing an enhancement in elasticity and fatigue resistance of 30,000 cycles at 50% compression strain. Nevertheless, despite this development, there still remains a demand for further investigation of biomass-derived precursor and its carbonization conditions to develop an all-biomass-derived carbon aerogel with super-elasticity and fatigue resistance under severe compression strains.

The rational design of microstructures is another attractive research avenue for developing elastic carbon aerogels.^12^ Specifically, anisotropic microstructure designs such as cellular-like,^20^ lamellar-like,^21^ wood-like,^22^ and honeycomb-like^3^ anisotropic microstructures could maximize the elasticity and fatigue resistance of the aerogels in the direction perpendicular to the anisotropic microstructures. Inspired by natural honeycombs and their anisotropic microstructures,^23^ it has been verified that honeycomb-structured aerogels exhibited apparent densities similar to those of typical aerogels^24^ but were structurally robust because the cell walls were closely connected and formed a “Steiner tree” geometry.^25^ This could efficiently dissipate mechanical strength and guide the deformation, thus avoiding locally large deformation and structural failure.^26^ For example, Ding et al. developed an elastic composite carbon aerogel with anisotropic “Steiner tree” microstructures based on carbonized konjac glucomannan and SiO_2_ nanofibers.^19^ Notably, the thermally stable and mechanically robust SiO_2_ nanofibrous networks entangled within the microstructures could be defoamed flexibly to dissipate mechanical strength and avoid the stress concentration, thereby imparting the composite carbon aerogel with super cyclable compressibility.^19^ However, the rational design of anisotropic microstructures with nanofibrous networks in carbonized biomass aerogels remains a challenge because bio-nanofibers frequently suffer from severe collapse of their original morphologies upon high-temperature carbonization.^27^

Cellulose and chitin nanofibers are the two most abundant bio-nanofibers on earth. While cellulose nanofibers inevitably collapse their original morphologies during high-temperature carbonization, chitin nanofibers reportedly exhibit higher morphological stability than cellulose nanofibers against carbonization.^27,28^ Furthermore, the high aspect ratios of chitin nanofibers^29^ can be beneficial to forming entangled nanofibrous networks. Therefore, chitin nanofibers (denoted as nanochitin) have the potential to be an ideal precursor for the fabrication of carbonized biomass aerogels with well-designed microstructures with nanofibrous networks. Although nanochitin-derived carbons have previously been reported,^30−34^ their fabrication conditions required to boost their elastic properties have not yet been systematically explored.

In this study, an all-nanochitin-derived carbon aerogel with honeycomb-like anisotropic microstructures and nanofibrous networks (denoted as nanochitin-derived carbon honeycomb) was fabricated from a nanochitin/water suspension via unidirectional freeze-drying and subsequent carbonization. The nanochitin concentration and carbonization temperature were optimized to impart the nanochitin-derived carbon honeycomb with super-compressibility, super-elasticity, and robustness. The optimized carbon honeycomb exhibited excellent elasticity as well as fatigue resistance even at a 90% compression strain. The elasticity of the carbon honeycomb was maintained even when exposed to flame and liquid nitrogen owing to its high thermal stability. Owing to these features, the carbon honeycomb offered high pressure sensitivity over an ultrawide range of working pressures and temperatures.

## 2 Experimental Section

### 2.1 Fabrication of the Nanochitin-Derived Carbon Honeycombs

**Figure 1:**
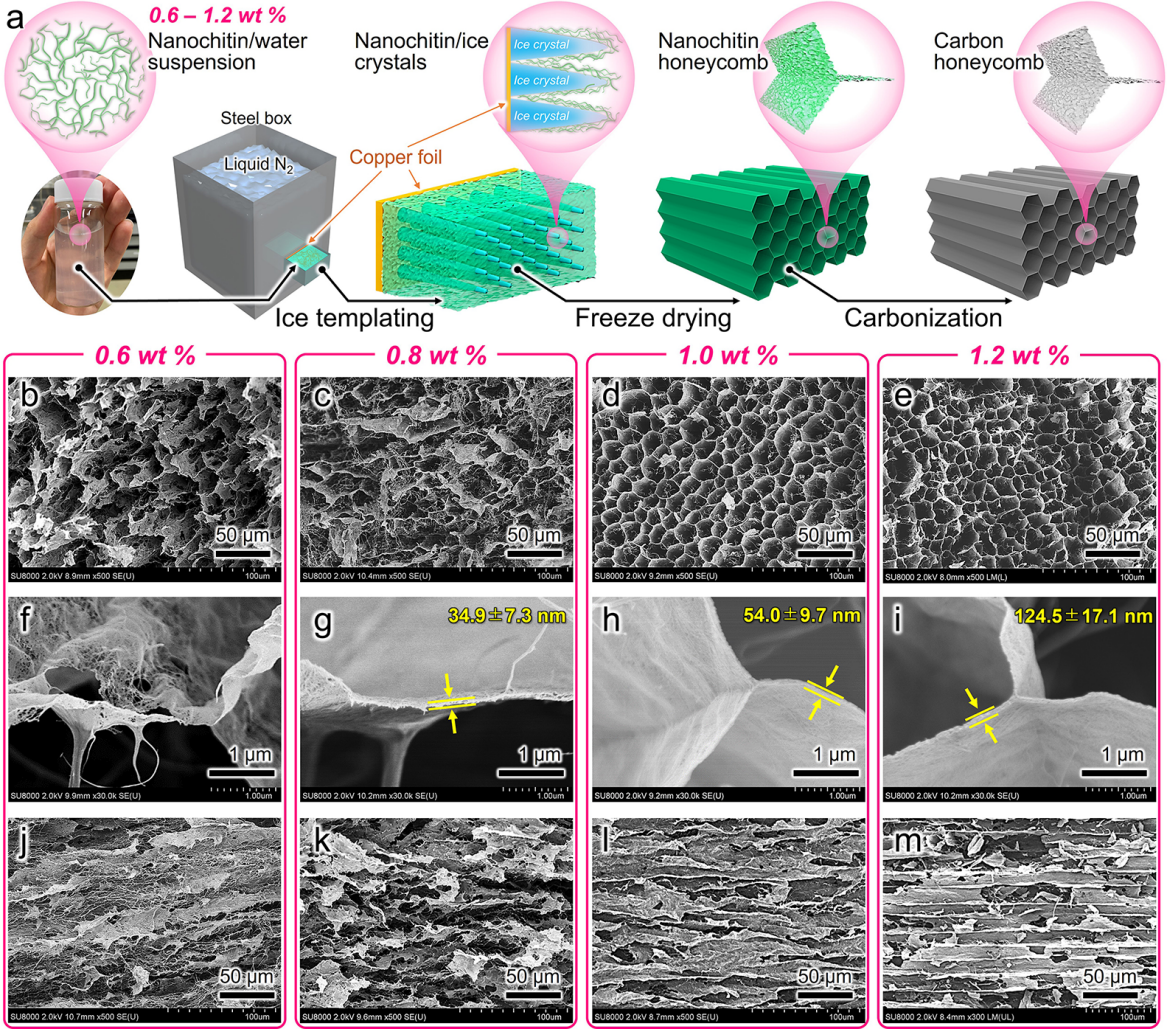
Fabrication and morphology of the nanochitin-derived carbon honeycombs. (a) Schematic of the preparation. FE-SEM images in the (b–i) parallel and (j–k) perpendicular directions of the anisotropic porous channels of the carbon honeycombs prepared at nanochitin concentrations of (b,f,j) 0.6, (c,g,k) 0.8, (d,h,l) 1.0, and (e,i,m) 1.2 wt %. Carbonization temperature: 900 °C.

Nanochitin-derived carbon honeycombs were fabricated via unidirectional ice-templating, freeze-drying, and subsequent carbonization (Figure 1a). Nanochitin/water suspensions (24 mL, BiNFi-s chitin, SFo-20002, Sugino Machine, Ltd., Namerikawa, Japan) with controlled nanochitin concentrations (0.6, 0.8, 1.0, and 1.2 wt %) were first defoamed using a centrifugal vacuum apparatus (ARV-930TWIN, Thinky Corp., Tokyo, Japan) at 1400 rpm at 25 °C for 5 min. The as-defoamed suspensions were then poured into boxes (length = 40 mm, width = 30 mm, depth = 20 mm) made of acrylic plates (thickness = 1.0 mm; AcrySunday Co. Ltd., Osaka, Japan); in each box, an inner wall (length = 40 mm, thickness = 20 mm) was pre-attached using adhesive Cu foil (no. 8701-00, Maxell Sliontec Ltd., Kanagawa, Japan). Subsequently, the nanochitin/water suspension-filled acrylic boxes (with the Cu side) were attached to a liquid nitrogen-filled open steel box for unidirectional ice-templating for 30 min, followed by freeze-drying below −90 °C and 5.0 Pa for 60 h using a freeze dryer (EYELA FDU-2200, Tokyo Rikakikai Co. Ltd., Tokyo, Japan). The resulting nanochitin-derived honeycombs were carbonized in a temperature-programmable muffle furnace (KDF75, Denken Highdental Co., Ltd. Kyoto, Japan) in three stages: (1) the honeycombs were heated from 25 to 500 °C at a heating rate of 2 °C min^–1^, and the temperature was maintained for 2 h; (2) the temperature was increased to the target carbonization temperature (600, 700, 800, 900, or 1000 °C) at a heating rate of 5 °C min^–1^ and maintained for 1 h; (3) the carbonized honeycombs were cooled to 25 °C at 2 °C min^–1^. Carbonization was performed in a nitrogen atmosphere.

### 2.2 Evaluation of the Elastic Properties of Nanochitin-Derived Carbon Honeycombs

The elastic properties of the nanochitin-derived carbon honeycombs were evaluated using a compression testing apparatus equipped with a 500 N load cell (EZ-SX, Shimadzu Co. Ltd., Osaka, Japan). The carbon honeycomb was first cut into a cuboidal shape (typical size: length = 15 mm, width = 15 mm, thickness = 10 mm) and set between the two compression stages; the top stage was moved to apply and release uniaxial compression force on the carbon honeycomb. The loading and unloading directions were perpendicular to the anisotropic channels of the carbon honeycombs. Hysteresis curves with maximum strains of 40, 60, 70, 80, and 90% were measured at a stroke rate of 10 mm min^–1^ at 25 °C. Elastic property measurements at ultrahigh and ultralow temperatures were also performed by exposing the carbon honeycombs to a butane blowlamp flame (approximately 1000 °C; Shinfuji Burner Co. Ltd., Aichi, Japan) and liquid nitrogen (approximately −196 °C), respectively. For the measurement of fatigue resistance during 10,000 cycles of 60, 70, 80, and 90% compression and release, continuous cyclic compression was performed on the carbon honeycomb with a compression frequency of 0.4 Hz using a Desktop Model Endurance Testing Machine (DLDM111LHB, YUASA System Co. Ltd. Okayama, Japan). The stress and height retention values of the carbon honeycombs were calculated by dividing the stress and height at each cycle by those of the original state.

### 2.3 Evaluation of the Pressure-Sensing Performance of Nanochitin-Derived Carbon Honeycombs

A carbon honeycomb-based pressure sensor was fabricated as follows. The carbon honeycomb was first cut into a cuboid (typical size: length = 15 mm, width = 15 mm, thickness = 10 mm) and sandwiched between two Cu foil electrodes that were pre-adhered to a glass plate (thickness = 1 mm; Matsunami Glass Ind., Ltd., Osaka, Japan). The pressure-sensing performance of the carbon honeycomb was evaluated using a universal compression testing machine (MCT-1150, AND Co., Ltd., Tokyo, Japan) and an electrochemical workstation (ModuLab XM, Solartron Analytical, AMETEK Advanced Measurement Technology Inc., Berkshire, UK) at an applied voltage of 1 V. The pressure-sensing performances of the sensor at ultrahigh and ultralow temperatures were measured while exposing the carbon honeycombs to an alcohol lamp flame (approximately 600 °C; Maruemu Corp., Osaka, Japan) and liquid nitrogen (approximately −196 °C), respectively. The sensor was sandwiched between Al_2_O_3_ plates (1-1736-01, AS ONE Corp., Osaka, Japan) to protect the compression testing machine from the flame.

The relative current change (Δ*I*/*I*_0_) upon applying pressure was calculated using eq 1.
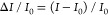
1where *I*_0_ (mA) is the initial current without pressure and *I* (mA) is the current at the applied pressure. The pressure sensitivity (*S*, kPa^–1^) was calculated using eq 2
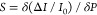
2where δ(Δ*I*/*I*_0_) is the change in Δ*I*/*I*_0_ and δ*P* (kPa) is the change in the applied pressure.

### 2.4 Characterization

The morphologies of the carbon honeycombs were observed using a field emission scanning electron microscopy (FE-SEM) machine (SU-8000, Hitachi High-Tech Science Corp., Tokyo, Japan) at an acceleration voltage of 2 kV. Prior to the FE-SEM observation, the carbon honeycombs were subjected to platinum sputtering at 20 mA for 10 s (ION SPUTTER, E-1045, Hitachi High-Tech Science Corp. Tokyo, Japan). The thicknesses of the channel walls of the carbon honeycombs were measured from the FE-SEM images using ImageJ software (USA). Elemental analysis was performed using an organic trace element analysis device (JM10, J-Science Lab Co., Ltd., Kyoto, Japan). FT-IR spectra were obtained using a KJP-05120S instrument (PerkinElmer Japan Co. Ltd., Kanagawa, Japan). The temperature was measured using a temperature meter (MT-309, MotherTool Co., Ltd. Nagano, Japan) with a wide-range temperature probe (Saveris T3, TESTO, SE & Co. KGaA, Titisee-Neustadt, Germany).

## 3 Results and Discussion

### 3.1 Preparation and Morphology of the Nanochitin-Derived Carbon Honeycombs

To realize nanochitin-derived carbon with nanofibrous networks and anisotropic microstructures similar to those of a honeycomb, a fabrication strategy is proposed based on the workflow illustrated in Figure 1a. Starting from a nanochitin/water suspension, the carbon honeycomb was fabricated via unidirectional ice-templating, freeze-drying, and subsequent carbonization. Nanochitin/water suspensions with concentrations of 0.6, 0.8, 1.0, and 1.2 wt % were prepared to tune the honeycomb microstructures. Owing to the unidirectional temperature gradient, the prism-shaped ice crystals grew vertically on the Cu foil and adjacent to one another. The adjacent ice crystals compel the nanochitin to form a continuous nanofibrous network as the honeycomb channel walls. After eliminating the prism-shaped ice crystals by freeze-drying, the nanochitin honeycomb with anisotropic honeycomb channels was obtained (Figure S1). Finally, the nanochitin honeycomb was carbonized at 900 °C to obtain the carbon honeycomb.

FE-SEM was performed to investigate the anisotropic honeycomb channels and channel walls of the carbon honeycomb. As shown in Figure 1b,c, at nanochitin concentrations lower than 0.8 wt %, the carbon honeycomb shows disorderly porous structures, implying that anisotropic honeycomb channels are hardly formed at low nanochitin concentrations. Upon increasing the nanochitin concentration to 1.0 wt %, anisotropic honeycomb channels are formed successfully (Figure 1d,e). Notably, the honeycomb channel walls are composed of entangled nanofibrous networks. It can be observed from the high-magnification FE-SEM images (Figure 1f–i) that when the nanochitin concentration is 0.6 wt %, numerous in-plane pores are formed within the nanofibrous networks (Figure 1f). With an increase in concentration from 0.8 to 1.2 wt %, the nanofibrous networks become densely stacked, with the stacking thickness increasing from 34.9 to 124.5 nm (Figure 1g–i); the detailed thickness distribution is summarized in Figure S2. In addition, highly ordered straight channels are observed in the side view of the carbon honeycomb (Figure 1j–m), indicating the successful formation of anisotropic honeycomb channels. Therefore, it was found that the microstructures varied with the nanochitin concentration. A concentration of more than 1.0 wt % is necessary for fabricating anisotropic honeycomb channels and nanofibrous network channel walls.

### 3.2 Elastic Properties of the Nanochitin-Derived Carbon Honeycombs Prepared at Different Nanochitin Concentrations

**Figure 2:**
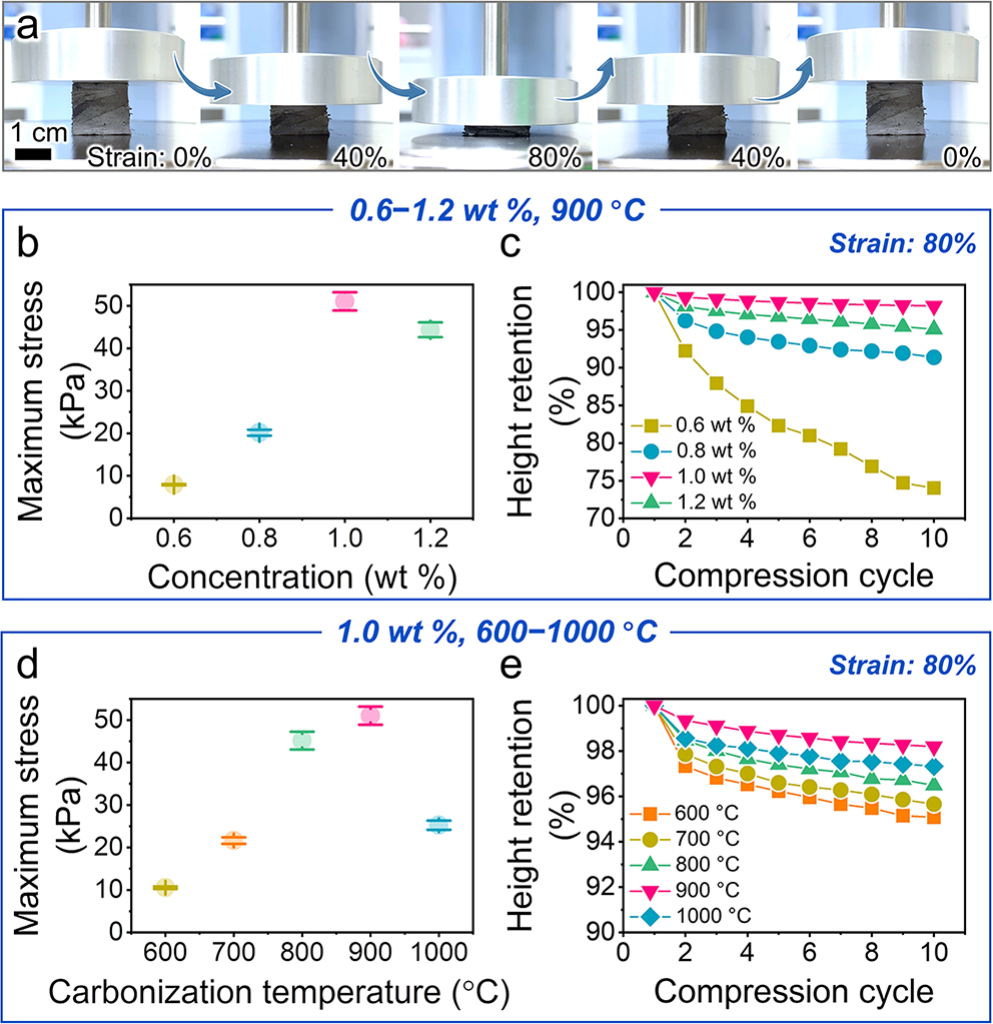
Elastic properties of the nanochitin-derived carbon honeycombs. (a) Optical images of the carbon honeycomb under strain ranging from 0 to 80% during compression loading and unloading. (b) Maximum stress and (c) height retention versus compression cycle of the carbon honeycombs prepared at varied nanochitin concentrations of 0.6, 0.8, 1.0, and 1.2 wt % and a carbonization temperature of 900 °C. (d) Maximum stress and (e) height retention versus compression cycle of the carbon honeycombs prepared at a nanochitin concentration of 1.0 wt % and varied carbonization temperatures of 600, 700, 800, 900, and 1000 °C.

To demonstrate the superiority of the designed anisotropic honeycomb channels and channel walls, the elastic properties of carbon honeycombs prepared at different nanochitin concentrations and a carbonization temperature of 900 °C are evaluated under successive cyclic compression up to 80% strain (Figure 2a). Notably, the chitin honeycomb that did not undergo carbonization does not deform elastically. The carbon honeycomb fabricated at a concentration lower than 0.8 wt % exhibits remarkable plastic deformation (Figure S3a,b) with a maximum stress of less than 20.0 kPa (Figure 2b) and a height retention of less than 91% (Figure 2c) after 10 compression cycles. This indicates that the carbon honeycombs without anisotropic honeycomb channels (Figure 1b,c) and sufficiently connected channel walls (Figure 1j,k) are too brittle to resist compression. In contrast, the carbon honeycomb with a 1.0 wt % nanochitin concentration exhibits an enhanced maximum stress of 51.0 kPa and height retention of 98.2% (Figure 2b,c), which indicates that the anisotropic honeycomb channels, which have wall thicknesses of approximately 54.0 ± 9.7 nm (Figure 1h), are robust but flexible to resist the compression and recover completely (Figure S3c). The carbon honeycomb with 1.2 wt % nanochitin concentration also shows a high-stress value, similar to that of the carbon honeycomb at 1.0 wt %, owing to the formation of anisotropic honeycomb channels. However, its retention decreases to 95.1% (Figure 2c), and brittle breakage occurs during cyclic compression (Figure S3d). This is because the thick channel walls (thickness = 124.5 ± 17.1 nm) (Figure 1i) with densely stacked carbon nanofibers suffer higher stress concentration, resulting in fragile breakage and poor elasticity.^15^ The nanochitin-derived carbon aerogel with random porous microstructures (without honeycomb-like anisotropic microstructures) also showed poor elasticity (Figure S4). Thus, elastic properties are realized for the nanochitin-derived carbon honeycomb tailored by tuning the anisotropic honeycomb channels and channel wall thicknesses by using a nanochitin concentration of 1.0 wt %.

### 3.3 Elastic Properties of the Nanochitin-Derived Carbon Honeycombs Prepared at Different Carbonization Temperatures

Based on a nanochitin concentration of 1.0 wt %, the effect of the carbonization temperature was further investigated to tailor the honeycomb channel walls and carbon-based molecular structures, which can optimize the elasticity of the carbon honeycomb. As shown in Figure 2d,e, the carbon honeycomb carbonized at 900 °C exhibits the highest stress and height retention after 10 compression cycles at 80% strain. The stress remains above 0 kPa until the strain reaches 0% in the unloading process (Figure S5a), indicating complete recovery.^5^ The carbon honeycombs carbonized at temperatures lower than 800 °C exhibit low maximum stress and height retention, where the stress is reduced to 0 kPa before the strain returns to 0% after 10 cycles of compression (Figure S5a), representing the poor elasticity and permanent plastic deformation at low carbonization temperatures. At a carbonization temperature of 1000 °C, the stress on the carbon honeycomb decreases significantly (Figures 2d and S5c).

**Figure 3:**
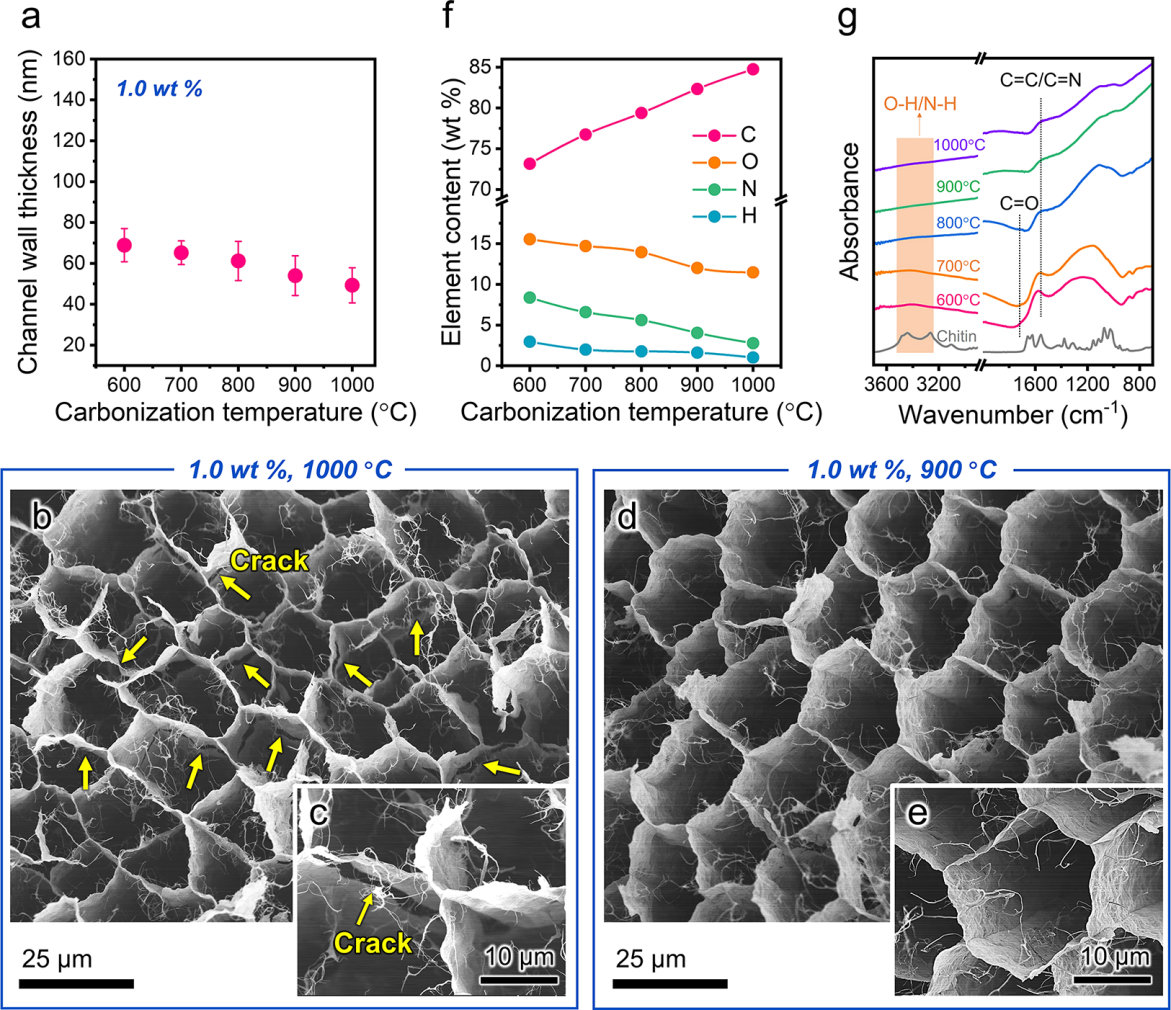
Honeycomb channel walls and their thickness and carbon-based molecular structure characterization for the nanochitin-derived carbon honeycombs. (a) Channel wall thickness of the nanochitin-derived carbon honeycombs as a function of carbonization temperature. FE-SEM images of the nanochitin-derived carbon honeycombs prepared at a nanochitin concentration of 1.0 wt % and varied carbonization temperatures of (b,c) 1000 °C and (d,e) 900 °C. (f) Elemental content and (g) FT-IR spectra of the nanochitin-derived carbon honeycombs prepared at a nanochitin concentration of 1.0 wt % and varied carbonization temperatures of 600, 700, 800, 900, and 1000 °C.

The elasticity of the nanochitin-derived carbon honeycombs upon carbonization can be understood by analyzing its honeycomb channel walls and their thicknesses and carbon-based molecular structures. As shown in Figures 3a and S6, when the nanochitin concentration is 1.0 wt %, the obtained honeycomb channel-wall thicknesses are approximately 68.9 ± 8.1, 65.2 ± 5.8, 61.2 ± 9.6, 54.0 ± 9.7, and 49.3 ± 8.6 nm at carbonization temperatures of 600, 700, 800, 900, and 1000 °C, respectively, implying that a gradual decrease in thickness might be a reason for the increase in elasticity with increasing carbonization temperature.^15^ However, the honeycomb carbonized at 1000 °C showed lower elasticity than that carbonized at 900 °C (Figures 2d,e and S5b,c). This is possibly due to the weight loss that occurs upon carbonization at 1000 °C, owing to which the density is only 5.0 mg cm^–3^ (Figure S5d), causing the honeycomb channel walls to crack (Figure 3b,c) after carbonization, thus resulting in weakened elasticity. In contrast, such cracks are not observed in the honeycomb carbonized at 900 °C (Figure 3d,e), showing good elasticity. These results suggest that a carbonization temperature of 900 °C is preferred for the production of elastic carbon honeycombs based on nanochitin, as a higher carbonization temperature of 1000 °C results in lower density of the carbon honeycomb. The nanochitin-derived honeycomb carbonized at 1000 °C with a 1.2 wt % nanochitin concentration has a similar density of 5.79 mg cm^–3^ to that of the honeycomb carbonized at 900 °C with a 1.0 wt % nanochitin concentration (5.72 mg cm^–3^) (Figure S5d). However, the honeycomb channel walls carbonized at 1000 °C are distorted (Figure S5e), which would also result in poor elasticity upon compression (Figure S5c).

For the carbon-based molecular structures, elemental analysis suggests that the C content increases from 73.15 to 84.75 wt % with increasing carbonization temperature from 600 to 1000 °C, while the O, N, and H contents gradually decrease from 15.54 to 11.47, 8.36 to 2.77, and 2.95 to 1.01 wt %, respectively, indicating that O, N, and H are gradually removed from the carbon honeycomb (Figure 3f). The Fourier-transform infrared (FT-IR) spectra show that the O–H and N–H characteristic peaks gradually decrease with increasing carbonization temperatures from 600 to 1000 °C (Figure 3g and Note S1), similar to the tendency for the enhancement of elasticity from 600 to 900 °C. Due to the gradual removal of the H, N, and O elements, the hydrogen bonding provided by O–H and N–H was significantly weakened after carbonization.^35^ This implies that the weak interactions between the carbonized nanochitin play an important role in enhancing the elasticity of the carbon honeycomb, as reported for cellulose nanofiber materials.^36,37^ Despite the hydrogen bonding interactions between the carbonized nanochitin being weak, the honeycomb carbonized at 1000 °C shows low elasticity due to the formation of cracks within its thin channel walls by the occurrence of severe weight loss.

Therefore, it is reasonable to conclude that the nanochitin-derived carbon honeycomb with a 1.0 wt % nanochitin concentration and 900 °C carbonization temperature has excellent elasticity owing to the desirable honeycomb-like microstructures with entangled nanofibrous networks, thin channel walls with fewer cracks, and weak interactions between carbonized nanochitin. The honeycomb-like microstructures with thin channel walls are mechanically robust, providing a structure with high compressibility and good recovery. The weakened interactions between the carbonized nanochitin effectively mitigate the stickiness of the carbon honeycomb, thus dissipating the compression stress concentration and guiding the deformation of the microstructures to avoid structural failure during compression and recovery.

### 3.4 Fatigue Resistance of the Nanochitin-Derived Carbon Honeycombs

**Figure 4:**
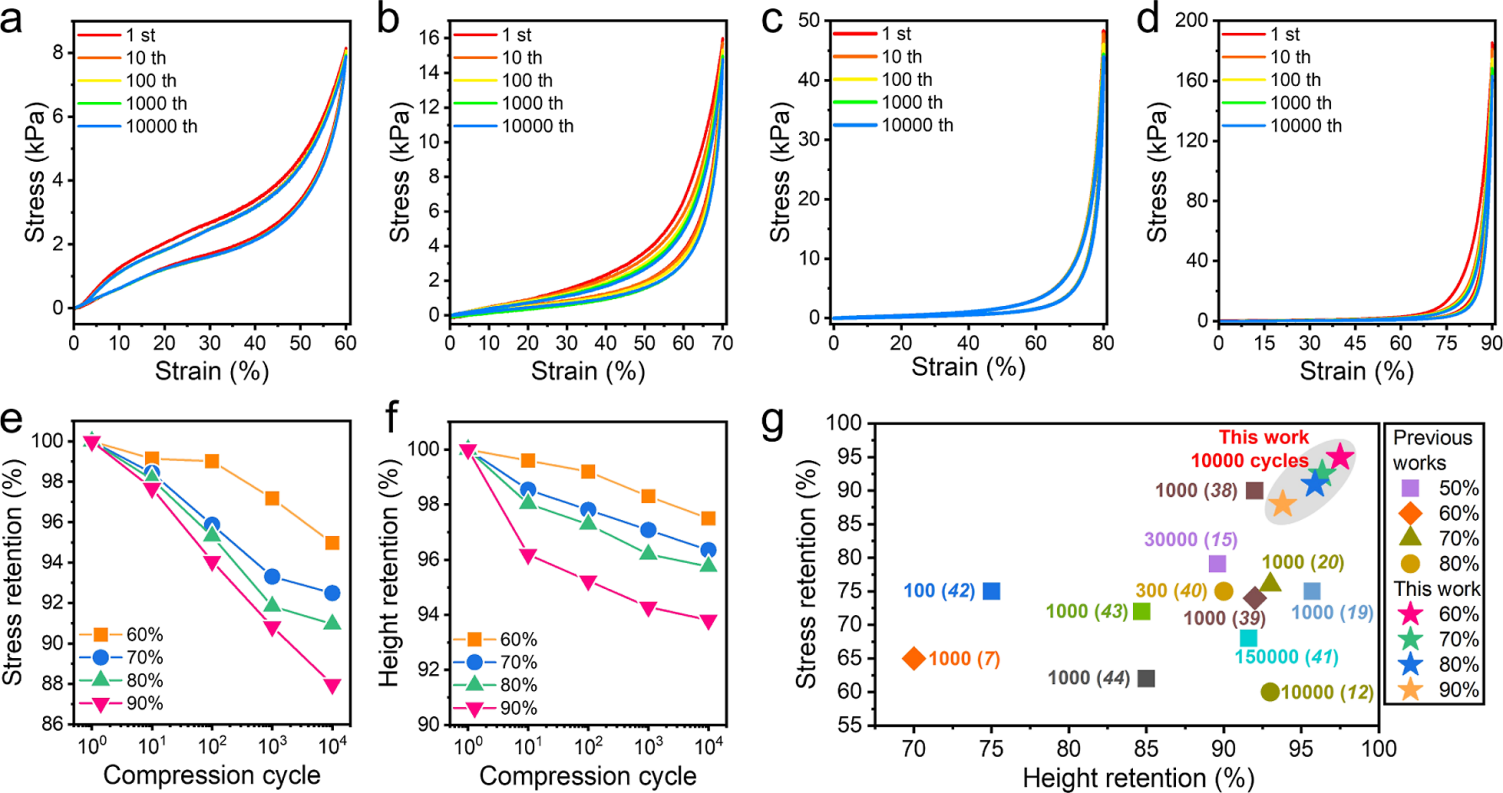
Fatigue resistance of the carbon honeycomb prepared at a nanochitin concentration of 1.0 wt % and a carbonization temperature of 900 °C. Stress–strain curves of the carbon honeycombs during compression loading and unloading at (a) 60, (b) 70, (c) 80, and (d) 90% strain for 10,000 cycles of compression. (e) Stress and (f) height retention during 10,000 cycles of compression at 60, 70, 80, and 90% strain. (g) Comparison of stress and height retention of nanochitin-derived elastic carbon honeycombs with previously reported carbon materials. Compression cycle numbers are included beside the corresponding dots in the chart. Numbers in parentheses refer to relevant references. The percentages shown in the legend represent the relevant compression strain for each study.

The optimized elastic carbon honeycomb was subjected to compression–release cycles at compression strains starting from 60% to test its fatigue resistance. As shown in Figure 4a, the carbon honeycomb showed a plateau region at compression strains of above ∼10% during the loading process, while the stress remained above 0 until the strain reached 0% during the unloading process, indicating almost no plastic deformation. Hence, this plateau region indicates the elastic buckling of the thin channel walls within the carbon honeycomb^12^ rather than its plastic deformation. The carbon honeycomb shows almost overlapping stress–strain curves at a maximum strain of 60%, confirming its elasticity over 10,000 cycles of compression. Notably, stress and height retentions of 95 and 97.5%, respectively, are achieved by the elastic carbon honeycomb after 10,000 cycles of compression at 60% strain (Figure 4e,f), indicating negligible changes in elasticity. After compression for 10,000 cycles at strain levels of 70 and 80% (Figure 4b,c), the carbon honeycomb maintains over 91% of the maximum stress and 96% of the maximum height (Figure 4e,f). These results illustrate that the carbon honeycomb can tolerate large elastic deformations (within 80% strain) without undergoing damage or structural collapse. Moreover, even under a more severe compression strain of 90% over 10,000 cycles (Figure 4d), the carbon honeycomb exhibits stress and height retentions of up to 87 and 94%, respectively; the height reduction occurs mainly in the earlier cycles, and the structure becomes relatively stable in the subsequent cycles (Figure 4f). As summarized in Figure 4g, the carbon honeycomb reported herein can exhibit excellent elasticity and fatigue resistance favorably comparable to those of state-of-the-art carbon aerogels, such as fossil-derived carbon aerogels,^7,20,38−40^ MXene aerogels,^41^ and biomass-based carbon composite aerogels.^12,15,19,42−44^ These results indicate the significance of the tailored microstructures and molecular structures within the carbon honeycomb.

### 3.5 Application of the Nanochitin-Derived Carbon Honeycombs in Pressure Sensing

**Figure 5:**
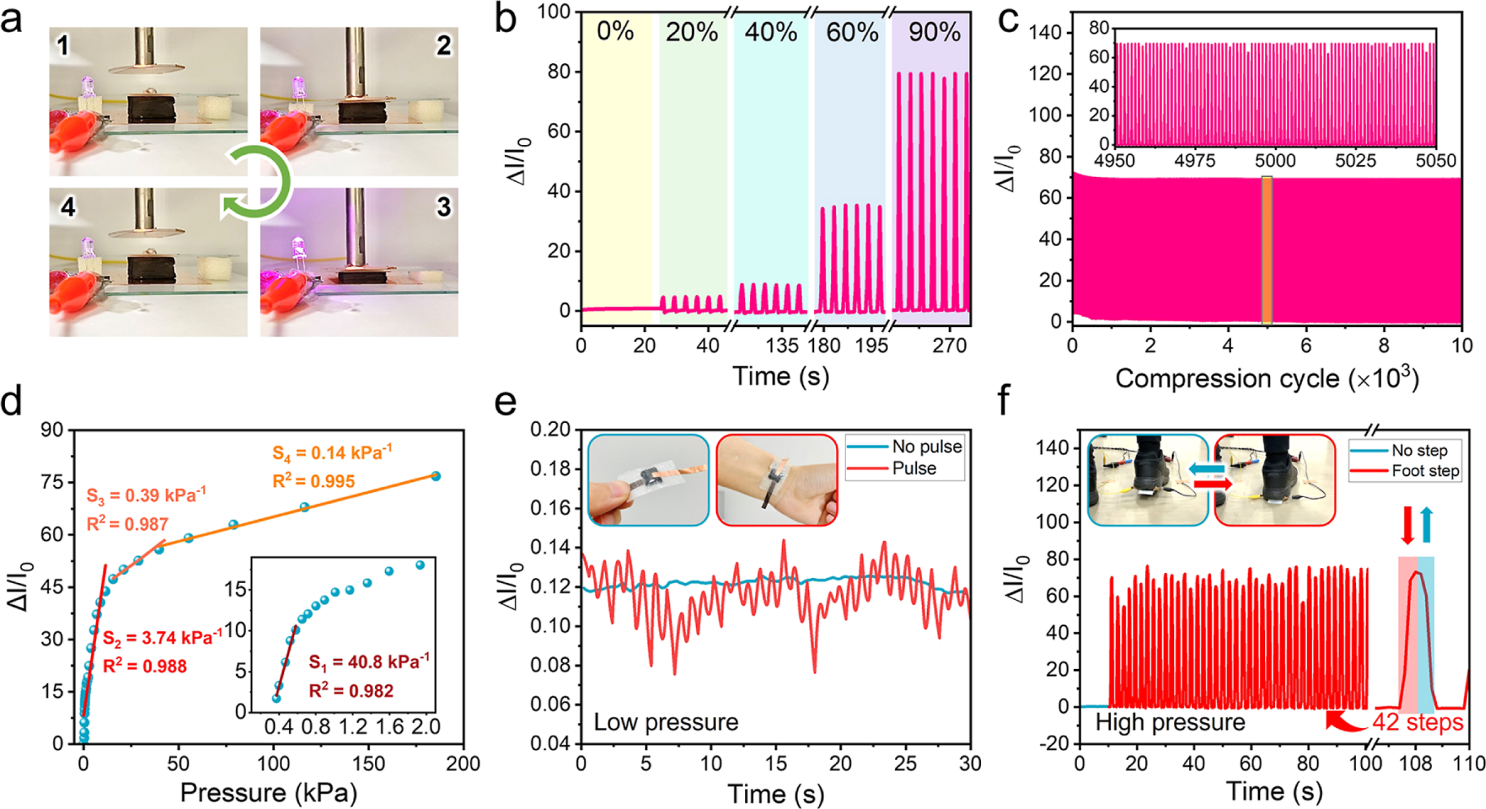
Pressure sensing performance of the nanochitin-derived carbon honeycomb at 25 °C. (a) The brightness of the LED is controlled by applying different pressures. (b) Relative current change (Δ*I*/*I*_0_) at different compression strains from 0 to 90%. (c) Δ*I*/*I*_0_ at a compression strain of 90% during 10,000 cycles. (d) Δ*I*/*I*_0_ and pressure sensitivity (*S*) over a wide pressure range from 0 to 185.5 kPa. Δ*I*/*I*_0_ of the carbon-honeycomb-based pressure sensor during the monitoring of human behavior: (e) low-pressure pulse vibration and (f) high-pressure footsteps. Nanochitin concentration: 1.0 wt %. Carbonization temperature: 900 °C.

The excellent compressibility, elasticity, and fatigue resistance of the nanochitin-derived carbon honeycomb at 90% strain make it a promising candidate for application in flexible electronics such as pressure sensors. Herein, the carbon honeycomb was applied to a pressure sensor. The pressure sensor was fabricated by sandwiching the carbon honeycomb between two Cu foil electrodes (Figure S7). As shown in Figure 5a, the real-time LED brightness achieved by the elastic carbon honeycombs changes with the compression strain, indicating that the electrical conductivity of the carbon honeycombs changes sensitively with compression. Figure 5b shows the real-time relative current change (Δ*I*/*I*_0_) of the carbon honeycombs for six or seven cycles at compression strains of 20, 40, 60, and 90%. Each peak corresponds to one cycle of compression and release. The Δ*I*/*I*_0_ increases considerably during compression and decreases rapidly during release, indicating the rapid current response of the elastic carbon honeycombs under compression. Furthermore, the maximum Δ*I*/*I*_0_ increases continuously with increasing pressure, demonstrating the potential applicability of the carbon honeycomb for pressure sensing. The current response of the elastic carbon-honeycomb-based pressure sensor remains at almost the same amplitude, without any substantial change after 10,000 cycles at 90% strain (Figure 5c), indicating its excellent current response stability. Figure 5d shows the calculated pressure sensitivity. Specifically, the elastic carbon honeycomb exhibits four linear pressure sensitivities of 40.8 kPa^–1^ (0–0.58 kPa), 3.74 kPa^–1^ (0.58–11.5 kPa), 0.39 kPa^–1^ (11.5–39.7 kPa), and 0.14 kPa^–1^ (39.7–185.5 kPa), demonstrating high sensitivity toward small pressure changes over a broad range of pressures.

Considering their exceptional sensitivity at both low and high pressures, the elastic carbon honeycombs were tested as wearable pressure sensors for detecting motions such as low-pressure pulse vibrations and high-pressure footsteps. As shown in Figure 5e, the Δ*I*/*I*_0_ value is stable when there is no pulse. By contrast, when detecting a pulse, the Δ*I*/*I*_0_ profile exhibits regular peaks with time, with each peak corresponding to a single pulse. A total of 33 pulse vibrations are recorded in 30 s, which is within the normal heart rate range for adults. In addition, Figure 5f shows that when the sensor is attached to the sole of a human foot, real-time current changes can be detected during walking. The minimum and maximum Δ*I*/*I*_0_ values for each step are stable, indicating that the sensor is stable under heavy foot compression. These results indicate that the elastic carbon honeycomb is applicable for the detection of motion in terms of both small and large pressure changes.

The pressure-sensing performance was demonstrated via current changes, which entailed monitoring the pressure-induced changes in the electrical conductivity of the elastic carbon honeycombs. The electrical conductivity of the carbon honeycombs depends on the point-to-point or point-to-plane contact numbers and the plane-to-plane contact area of the anisotropic honeycomb channels, which are elucidated as the following three key points: (i) in low-pressure regions, the electron conduction paths from one side to the other increase rapidly in the point-to-point or point-to-plane form^45^ owing to the abundant free carbonized nanochitin on the channel walls (Figure S8), thus imparting a high sensitivity (*S*_1_: 40.8 kPa^–1^) to pressure changes; (ii) in medium-pressure regions, the electron conduction paths are gradually converted to the point-to-plane and plane-to-plane forms,^46^ which are dominated by the contact area between the channel walls. With an increase in the contact area, change in the electron conductivity with increasing pressure gradually decreases, thus exhibiting a decreased pressure sensitivity (*S*_2_: 3.74 kPa^–1^); and finally, (iii) in high-pressure regions, the electron conduction path is mainly determined by the plane-to-plane contact area,^47^ wherein almost all channel walls are in contact with each other. As a result, the rate of variation of the plane-to-plane contact area between the channel walls decreases significantly, whereas the pressure variation rate drastically increases, weakening pressure sensitivity (*S*_3_: 0.39 kPa^–1^ and *S*_4_: 0.14 kPa^–1^).

**Figure 6:**
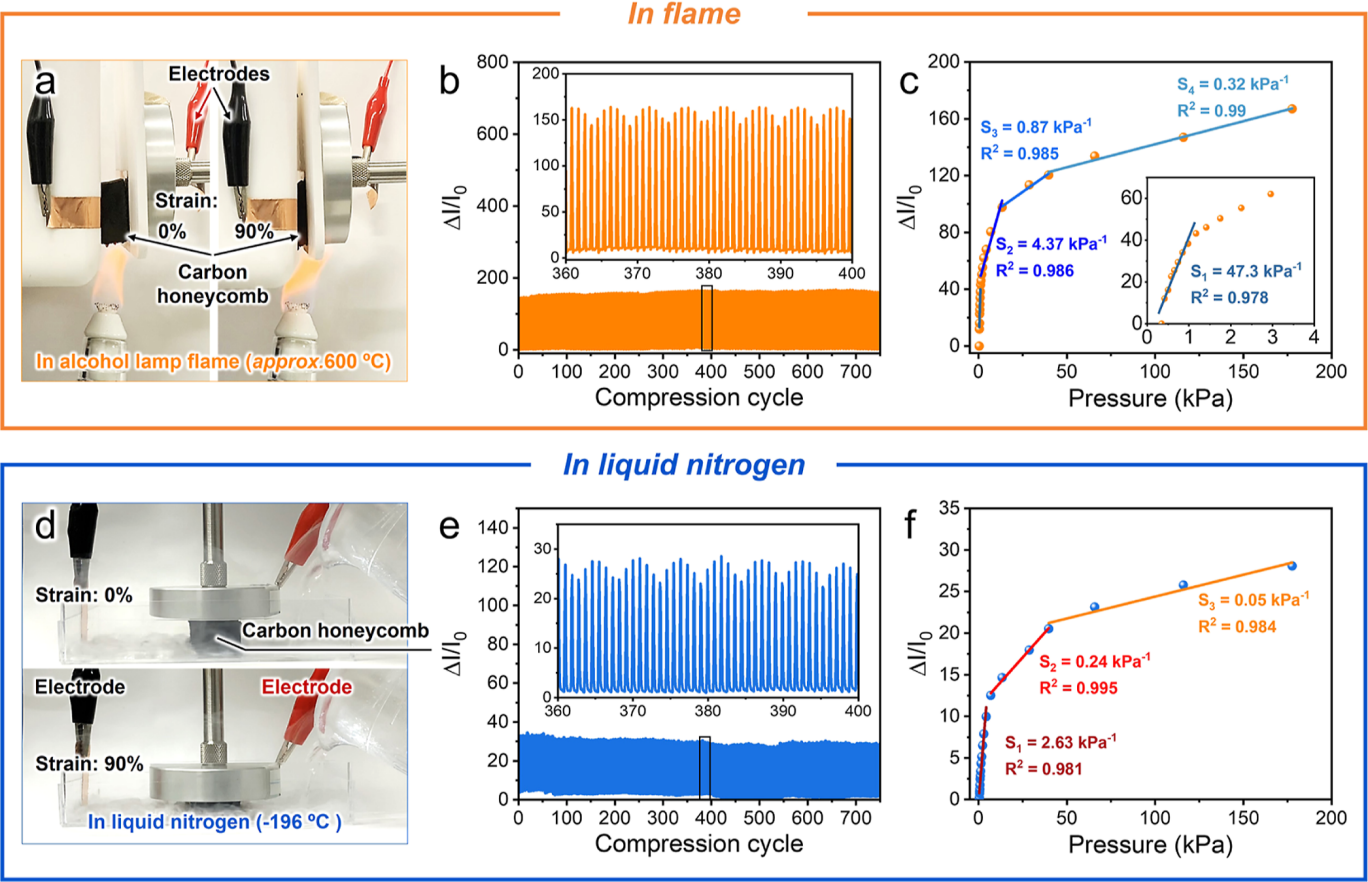
Pressure-sensing performance of the nanochitin-derived carbon honeycombs over an ultrawide temperature range. (a,d) Optical images, (b,e) Δ*I*/*I*_0_ at a compression strain of 90% during 750 cycles, and (c,f) Δ*I*/*I*_0_ and pressure sensitivity (*S*) over a wide pressure range from 0 to 185.5 kPa in (a–c) alcohol lamp flame and (d–f) liquid nitrogen. Nanochitin concentration: 1.0 wt %. Carbonization temperature: 900 °C.

The as-designed carbon honeycomb exhibits elasticity over an ultrawide temperature range, even when exposed to flame and liquid nitrogen (see Figures S9 and S10 and Video S1 and S2 for more details). To demonstrate this, the carbon honeycomb was used as a pressure sensor under severe conditions. The pressure-sensing properties were measured by assembling a sensor with thermal-stable components and exposing it to alcohol lamp flame (Figure 6a) and liquid nitrogen (Figure 6d), the temperatures of which were approximately 600 and −196 °C, respectively.

In the flame, the Δ*I*/*I*_0_ of the sensor maintains almost the same amplitude without significant changes over 750 compression cycles at a maximum strain of 90% (Figure 6b), indicating its pressure-sensing stability. Moreover, the pressure sensitivity in flame was measured via an in situ pressure-current recording system. The sensor demonstrates thermal stability and super elasticity in flame, exhibiting four linear pressure sensitivities of 47.3 kPa^–1^ (0–1.17 kPa), 4.37 kPa^–1^ (1.17–6.52 kPa), 0.87 kPa^–1^ (6.52–40.1 kPa), and 0.32 kPa^–1^ (40.1–177.6 kPa) (Figure 6c), which are comparable to those at 25 °C. This result indicates that the pressure sensor based on the nanochitin-derived carbon honeycombs is reliable, even when exposed to a flame.

In liquid nitrogen, the maximum Δ*I*/*I*_0_ decreases (Figure 6e). The sensor current at 0% strain was 42 and 5.3 mA in flame (600 °C) and liquid nitrogen (−196 °C), respectively, at a constant voltage of 1 V, suggesting that the electrical conductivity of the carbon honeycomb decreased with decreasing temperatures due to its semiconducting property (electrical conductivity at room temperature: 1.0 × 10^–2^ S cm^–1^). Thus, the sensor showed a lower Δ*I*/*I*_0_ value in liquid nitrogen than in flame due to the lower electrical conductivity of the carbon honeycomb in liquid nitrogen. However, the sensor still affords a stable current amplitude upon cyclic compression and release, verifying its pressure-sensing performance. Furthermore, three linear pressure sensitivities of 2.63 (0–4.27 kPa), 0.24 (4.27–38.5 kPa), and 0.04 kPa^–1^ (38.5–172.3 kPa) are obtained (Figure 6f), demonstrating its applicability for pressure sensing in ultralow temperatures. Such results confirm the potential for the application of the developed nanochitin-derived elastic carbon honeycombs over an ultrawide temperature range, such as pressure sensing in outer space.

The high pressure-sensing performance of the nanochitin-derived carbon honeycomb in flames and liquid nitrogen is related to its thermally stable elastic properties and tunable electrical conductivity upon compression. The elasticity even in flames and liquid nitrogen has been reported for ceramic sponges^48^ and CNT materials^49^ owing to their high thermal stability. The nanochitin-derived carbon honeycomb was also thermally stable even against such harsh conditions. Moreover, the electrical conductivity of the carbon honeycomb changed with the changes in pressure during compression and release, varying the current values and thus demonstrating pressure-sensing performance.

## 4 Conclusions

A nanochitin-derived elastic carbon honeycomb with honeycomb-like anisotropic microstructures was successfully fabricated via unidirectional ice templating, freeze-drying, and carbonization. A carbon honeycomb was optimized with a nanochitin concentration of 1.0 wt % and a carbonization temperature of 900 °C, which yielded ideal anisotropic honeycomb channels with entangled nanofibrous networks, thin channel walls with few cracks, and weak interactions between the carbonized nanochitin; this resulted in super-elasticity and high compressibility with up to 90% strain as well as stress and height retentions of 87 and 94%, respectively, after 10,000 cycles of compression. These metrics were better than those of most reported carbon aerogels derived from fossil fuels, biomass, and their composite materials. In addition, an as-tailored carbon honeycomb exhibited high thermal resistance and compression resilience from −196 to 1000 °C. A high-performance pressure sensor based on the carbon honeycomb demonstrated wide-range pressure detection for various motions. Furthermore, pressure sensitivity was ensured even in flame (600 °C) and in liquid nitrogen (−196 °C). These outstanding elastic, thermal, and electrical properties can potentially enable versatile applications in the future. Notably, our carbon honeycomb was fabricated from only biomass (nanochitin), whereas the conventional biomass-based elastic carbon aerogels have been mostly fabricated by compositing with fossil-derived carbons. A further challenge remains to fabricate the carbon honeycomb in a larger scale within a shorter time. This study breaks new ground in fabricating all-biomass-derived, super-elastic, and fatigue-resistant carbon materials for diverse applications.

## Supporting Information Available

FE-SEM image of chitin nanofibers and optical images of a nanochitin honeycomb and nanochitin-derived carbon honeycombs; FE-SEM images and channel wall thickness distribution of nanochitin-derived carbon honeycombs prepared at different nanochitin concentrations; stress–strain curves during loading and unloading at an 80% strain of nanochitin-derived carbon honeycombs prepared at different nanochitin concentrations; FE-SEM images of a nanochitin-derived carbon aerogel with random porous structures and optical images and stress–strain curves of the carbon aerogel with random porous structures with 10 compressions of loading and unloading at 80% strain; I–V curves of a nanochitin-derived carbon honeycomb-based pressure sensor over a wide voltage range under various pressures; FE-SEM image of a nanochitin-derived carbon honeycomb in a direction parallel to the anisotropic honeycomb channels and schematic representation of the deformation of the honeycomb structures and increased numbers of electron conduction pathways during compression and release; and optical images of a nanochitin-derived carbon honeycomb during compression and release in butane blowlamp flame and liquid nitrogen (PDF)
Nanochitin-derived carbon honeycomb during compression and release before, during, and after butane blowlamp flame treatment (MP4)
Nanochitin-derived carbon honeycomb during compression and release before, during, and after liquid nitrogen treatment (MP4)
