# Supplementary material for: All-Nanochitin-Derived, Super-Compressible, Elastic, and Robust Carbon Honeycombs and Their Pressure-Sensing Properties over an Ultrawide Temperature Range
Source: ACS Appl Mater Interfaces. 2023 Aug 23;15(35):41732–42. doi: 10.1021/acsami.3c08587 (PMC10485799; doi:10.1021/acsami.3c08587)
Supplement: Supplementary file 1 — am3c08587_si_001.pdf [file am3c08587_si_001.pdf]

Supporting Information

All-nanochitin-derived, super-compressible, elastic, and robust carbon honeycomb and its pressure sensing properties over an ultrawide temperature range

Xiang Li, Luting Zhu, Takaaki Kasuga, Masaya Nogi, Hirotaka Koga*

SANKEN (The Institute of Scientific and Industrial Research), Osaka University, 8-1 Mihogaoka, Ibaraki, Osaka 567-0047, Japan.

**Corresponding Author*

Hirotaka Koga

E-mail: hkoga@eco.sanken.osaka-u.ac.jp

Fax: +81-6-6879-8444; Tel: +81-6-6879-8442

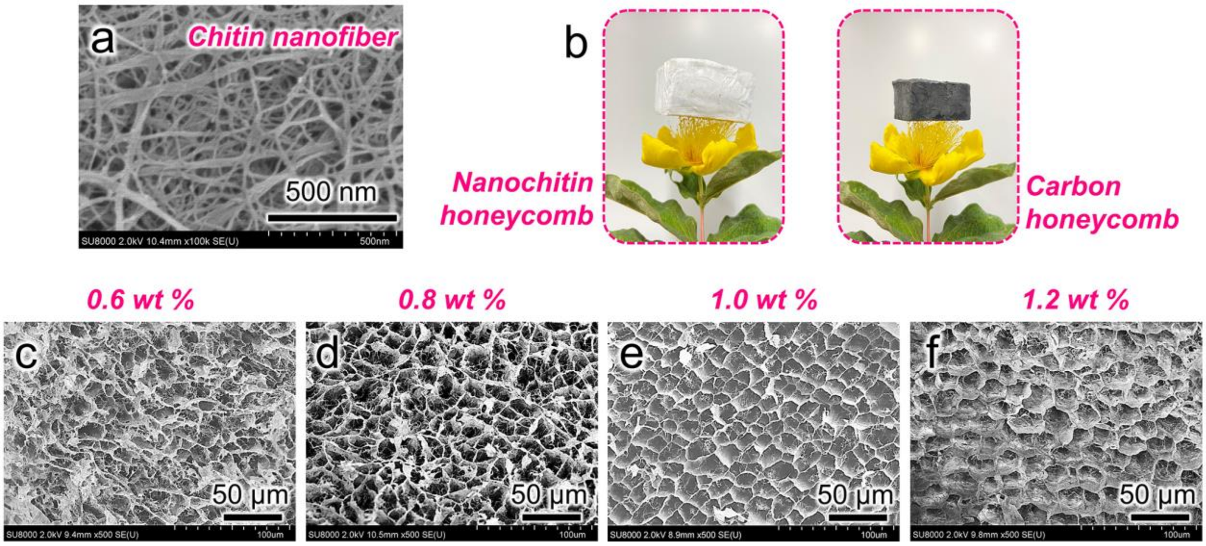

Figure S1. (a) An FE-SEM image of chitin nanofibers (denoted as nanochitin). (b) Optical images of nanochitin honeycomb and nanochitin-derived carbon honeycombs. FE-SEM images of chitin honeycombs prepared at varied nanochitin concentrations of (c) 0.6, (d) 0.8, (e) 1.0, and (f) 1.2 wt %.

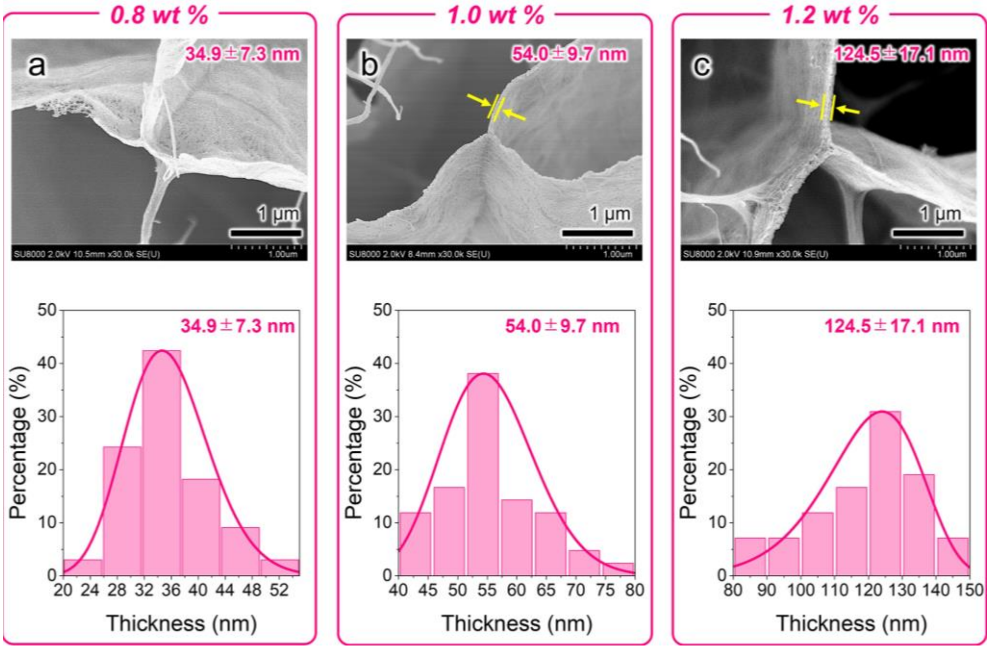

Figure S2. FE-SEM images and channel-wall thickness distribution of nanochitin-derived carbon honeycombs prepared at varied nanochitin concentrations of (a) 0.8, (b) 1.0, and (c) 1.2 wt % and a carbonization temperature of 900 °C.

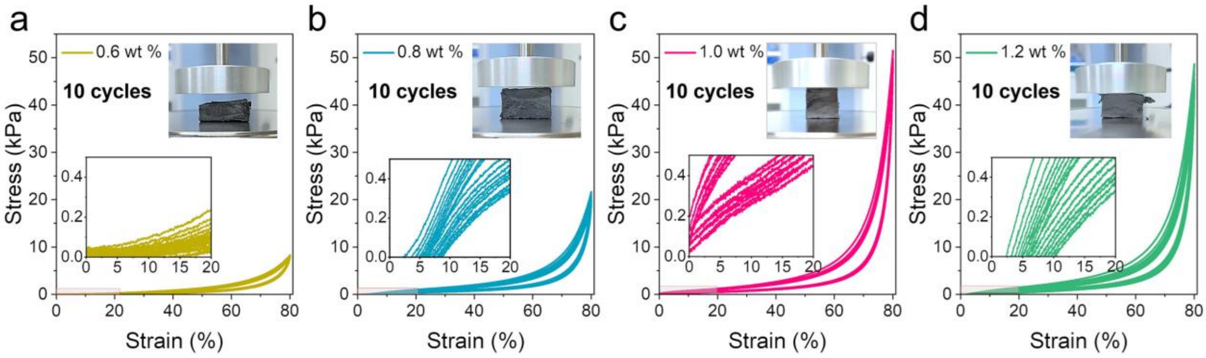

Figure S3. Stress–strain curves during compression loading and unloading at a strain of 80% for nanochitin-derived carbon honeycombs prepared at nanochitin concentrations of (a) 0.6, (b) 0.8, (c) 1.0, and (d) 1.2 wt % and a carbonization temperature of 900 °C.

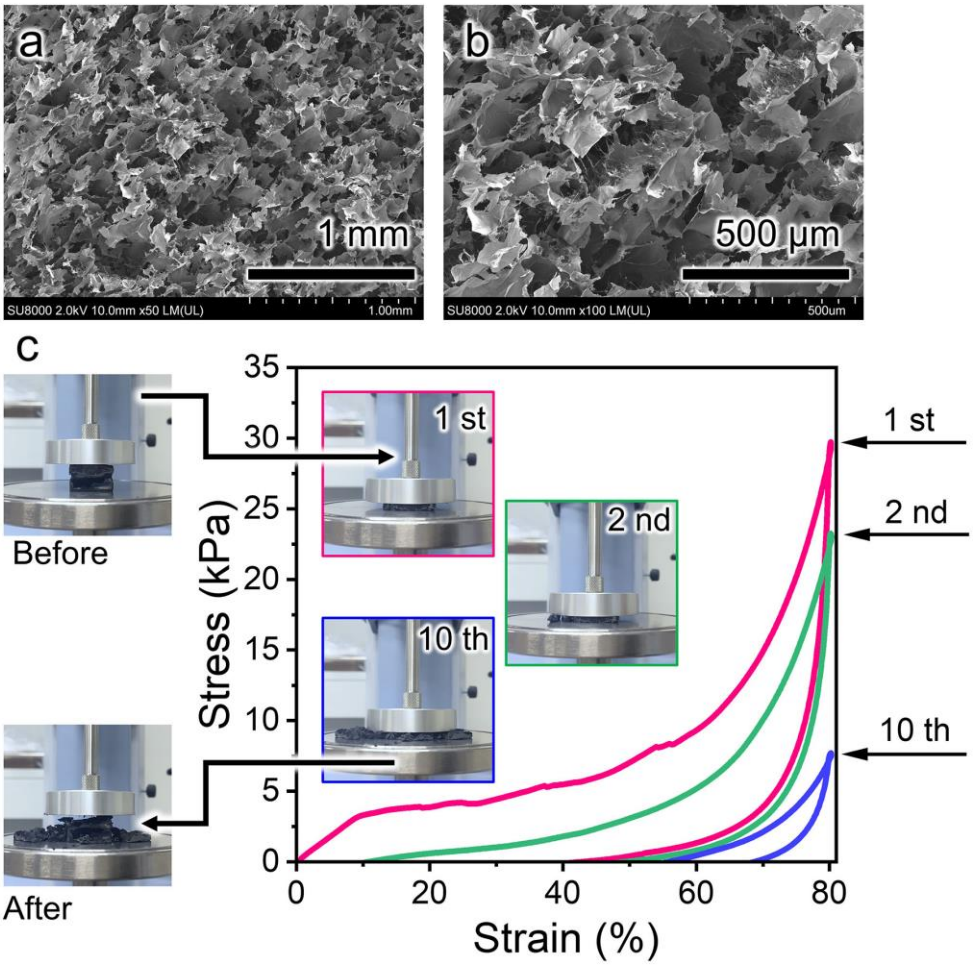

Figure S4. (a–b) FE–SEM images of the nanochitin-derived carbon aerogel with random porous structures. (c) Optical images and stress-strain curves of the carbon aerogel with random porous structures during 10 cycles of compression loading and unloading at 80% strain. Nanochitin concentration: 1.0 wt %. Carbonization temperatures: 900 °C.

The nanochitin-derived carbon aerogel with random porous microstructures was fabricated at a nanochitin concentration of 1.0 wt % and a carbonization temperature of 900 °C without using a unidirectional ice-templating method (**Figure S4a,b**). The carbon aerogel with random porous microstructures showed severe collapse and stress reduction during only 10 cycles of compression loading and unloading at 80% strain (**Figure S4c**), indicating its poor elastic properties.

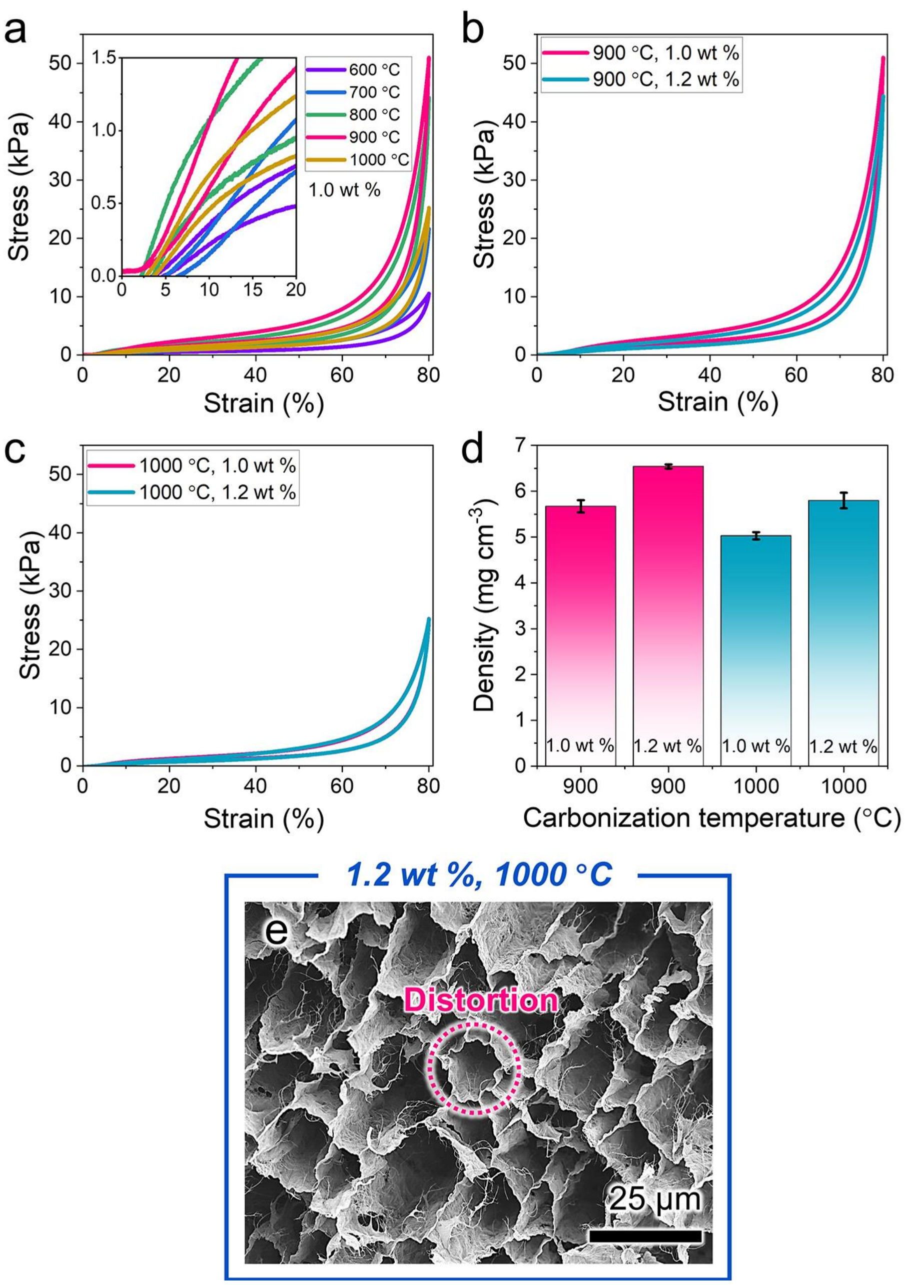

Figure S5. Stress–strain curves during compression loading and unloading at a strain of 80% for nanochitin-derived carbon honeycombs prepared at: (a) a nanochitin concentration of 1.0 wt % and varied carbonization temperatures of 600, 700, 800, 900, and 1000 °C, (b) varied nanochitin concentrations of 1.0 and 1.2 wt % and a carbonization temperature of 900 °C, and (c) varied nanochitin concentrations of 1.0 and 1.2 wt % and a carbonization temperature of 1000 °C. (d) Density of the carbon honeycombs prepared at varied nanochitin concentrations of 1.0 and 1.2 wt % and varied carbonization temperatures of 900 and 1000 °C. (e) An FE-SEM image of the carbon honeycomb prepared at a nanochitin concentration of 1.2 wt % and a carbonization temperature of 1000 °C.

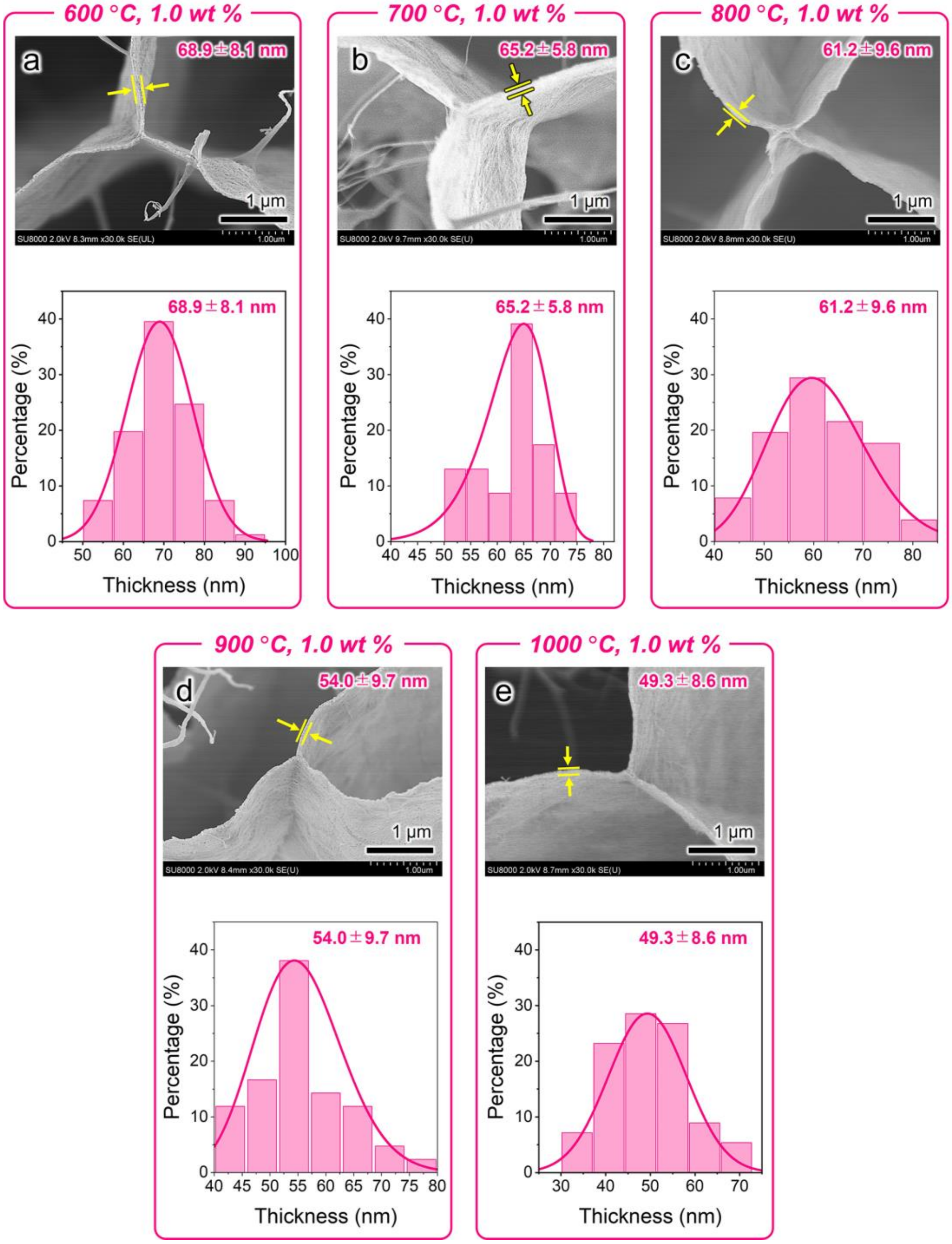

Figure S6. FE-SEM images and channel wall thickness distribution of nanochitin-derived carbon honeycombs prepared at a nanochitin concentration of 1.0 wt % and varied carbonization temperatures of (a) 600, (b) 700, (c) 800, (d) 900, and (e) 1000 °C.

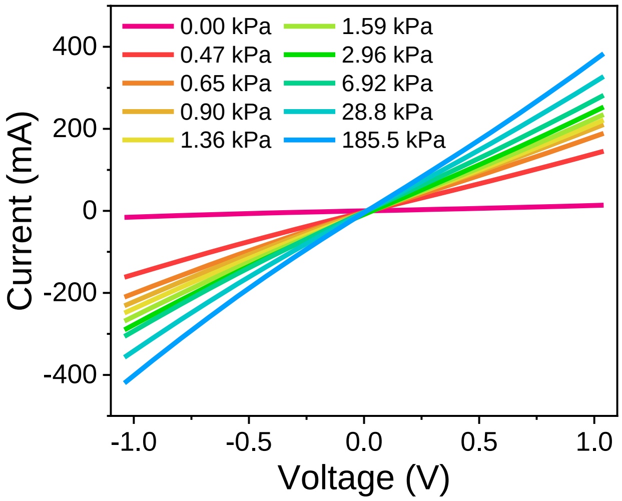

Figure S7. I–V curves of the nanochitin-derived carbon honeycomb-based pressure sensor over a voltage range of –1 V to 1 V under various pressures. Nanochitin concentration: 1.0 wt %. Carbonization temperature: 900 °C.

The pressure sensor was fabricated by sandwiching the carbon honeycomb between two Cu foil electrodes. The I–V curves of the pressure sensor were linear under various pressures, indicating Ohmic contact characteristics between the carbon honeycomb and the Cu foil electrodes.

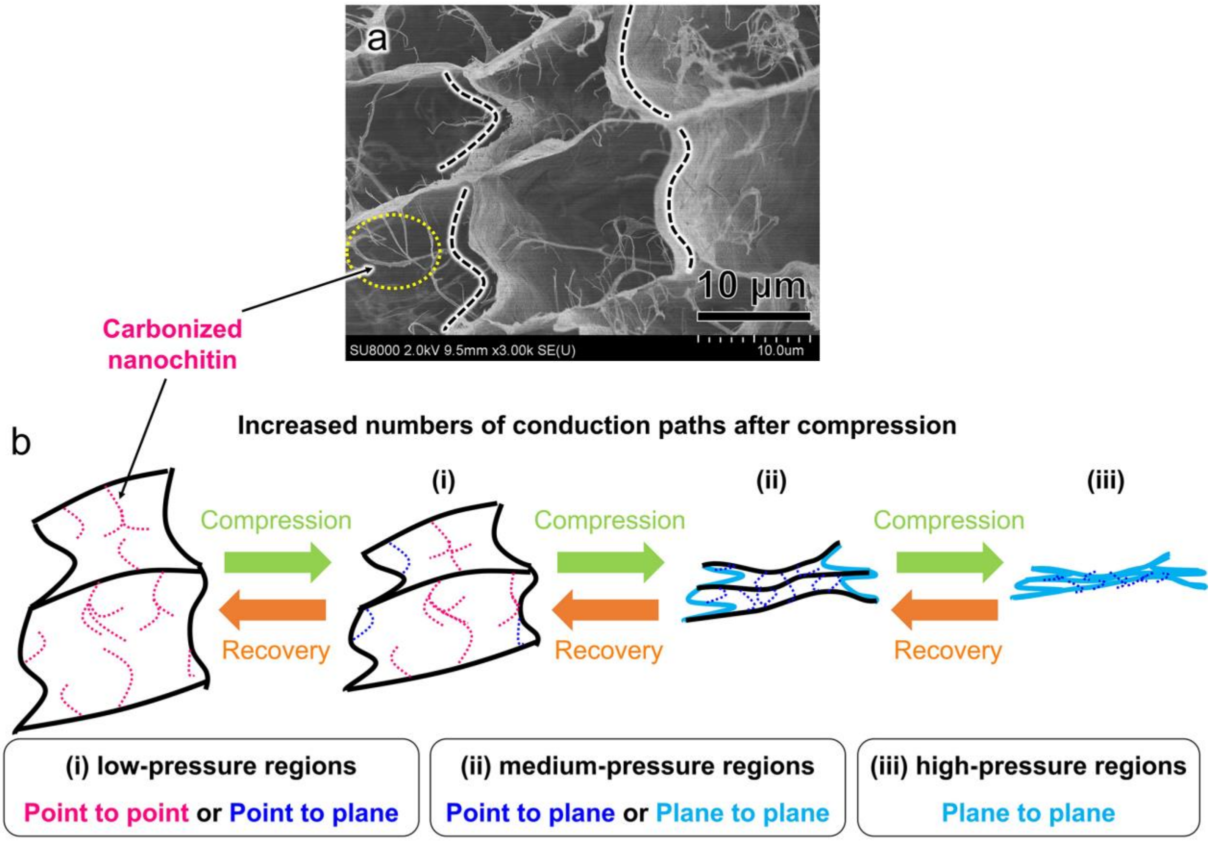

Figure S8. (a) An FE-SEM image of the nanochitin-derived carbon honeycomb in the direction parallel to the anisotropic honeycomb channels, showing the channel walls and free fibers outside them. (b) Schematic representation of the deformation of the honeycomb structures and increased numbers of electron conduction pathways during compression and release. Nanochitin concentration: 1.0 wt %. Carbonization temperature: 900 °C.

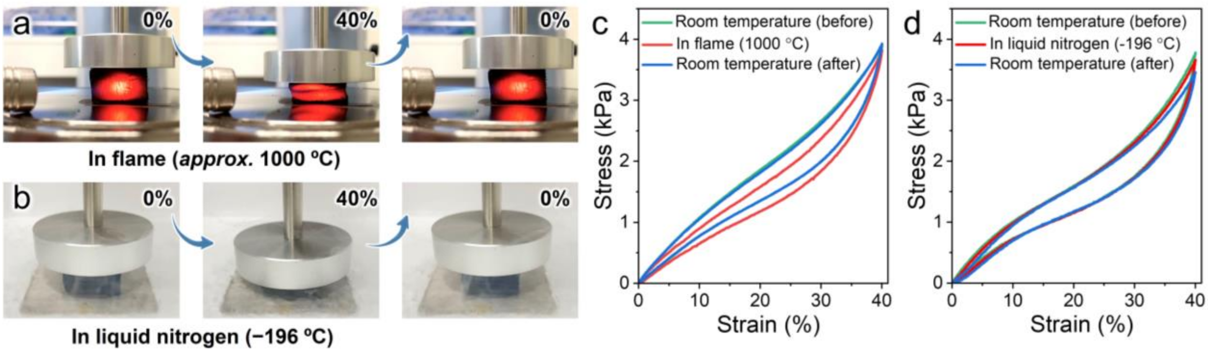

Figure S9. Optical images of the nanochitin-derived carbon honeycomb during compression and release in (a) butane blowlamp flame and (b) liquid nitrogen. Stress–strain curves of the carbon honeycomb before, during, and after (c) butane blowlamp flame and (d) liquid nitrogen treatment. Exposure time to flame (Approx. 1000 °C) and liquid nitrogen (−196 °C): 5 min. Nanochitin concentration: 1.0 wt %. Carbonization temperature: 900 °C.

Excellent resistance to thermal shock is vital for the practical application of electronic devices in extreme environments such as ultra-high- or ultra-low-temperature environments. In this study, we explored the elastic properties of an optimized nanochitin-derived carbon honeycomb over a wide temperature range, such as in flames and liquid nitrogen. As shown in **Figure S9a** and **Figure S10**, the carbon honeycomb exhibited good fire resistance against the flames of a butane blowlamp (Approx. 1000 °C) and an alcohol lamp (Approx. 600 °C). An in situ compression test was performed to evaluate the elasticity of the carbon honeycomb in the flame (**Video S1**). The maximun compression was set at 40% strain to avoid exposing the compression testing machine to the flame. No significant stress reduction was observed when the carbon honeycomb was exposed to flame (Approx. 1000 °C) for at least 5 min (during at least 30 compression cycles). Furthermore, when the carbon honeycomb was recovered to room temperature, the stress–strain curve overlapped with the original curve (**Figure S9c**). This indicates the thermal stability of the elastic carbon honeycomb against the high temperature treatment (Approx. 1000 °C) for at least 5 min. When the carbon honeycomb was placed in liquid nitrogen (−196 °C) (**Figure S9b**), it exhibited compressive and elastic behavior (**Video S2**) and could be compressed without obvious fracture and be recovered to its original shape. Notably, there was no significant deterioration in the mechanical properties of the carbon honeycombs compressed in liquid nitrogen for at least 5 min (during at least 30 compression cycles) (**Figure S9d**), illustrating their excellent elasticity at low temperatures.

These results suggest that the nanochitin-derived carbon honeycomb exhibits elasticity even in flame and liquid nitrogen. The elasticity in the flames was possibly due to the high thermal stability of the carbonized chitin molecular structure; thus, the honeycomb-like anisotropic porous structures could be preserved, resulting in elastic deformation. The elasticity in liquid nitrogen would be owing to hydrophobicity (low water content) as well as high thermal stability of the carbon honeycomb.

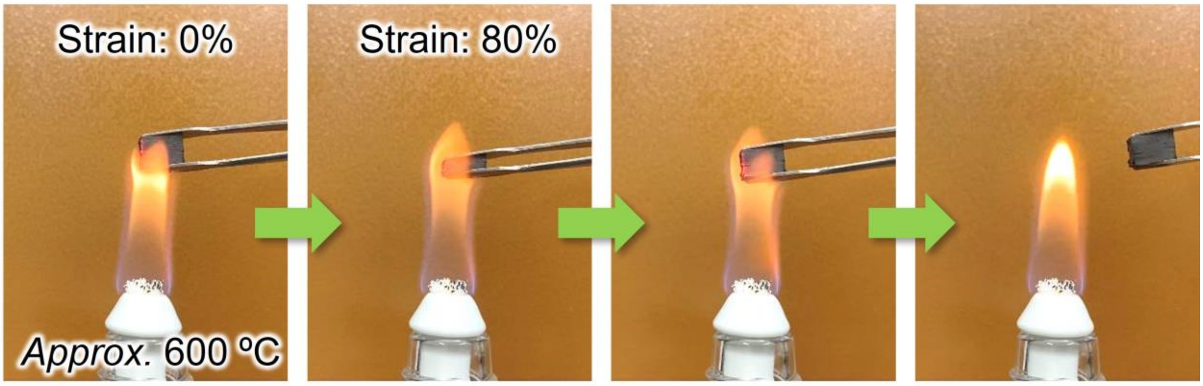

Figure S10. Optical images of the nanochitin-derived carbon honeycomb during compression and release in alcohol lamp flame with a temperature of approximately 600 °C. Nanochitin concentration: 1.0 wt %. Carbonization temperature: 900 °C.

Video S1

>Video of the nanochitin-derived carbon honeycomb during compression and release before, during, and after butane blowlamp flame treatment.

Video S2

> Video of the nanochitin-derived carbon honeycomb during compression and release before, during, and after liquid nitrogen treatment.

Note S1

To explain the hydrogen-bonding effect of the nanochitin-derived carbon honeycombs carbonized at different temperatures, Fourier-transform infrared spectroscopy (FT-IR) was performed (**Figure 3g**). The nanochitin honeycomb before carbonization showed peaks at 1660 and 1620 cm^{-1} corresponded to the amide I (C=O) region and those at 1560 cm^{-1} corresponded to the amide II (C–N) region, which are the characteristic of α-chitin.[1] The peaks at around 3450 and 3260 cm^{-1} were assigned to O–H and N–H stretching vibrations, respectively. After carbonization, the relative intensities of the O–H and N–H stretching vibrations of the carbon honeycomb gradually decreased with increasing temperature from 600 to 1000 °C, suggesting that the hydrogen bonding interactions were weakened owing to the loss of the O–H and N–H functional groups.[2] Meanwhile, the amide I and II peaks disappeared, and a new characteristic peak appeared at around 1580 cm^{-1} that corresponded to the vibrations of C=C and C=N [3] and was attributed to the formation of quinone structures,[4] indicating the formation of graphitic carbon structures upon carbonization.

References

(1) Goodrich, J. D.; Winter, W. T. α-Chitin Nanocrystals Prepared from Shrimp Shells and Their Specific Surface Area Measurement. *Biomacromolecules* **2007**, *8* (1), 252–257.

(2) Valentin, R.; Bonelli, B.; Garrone, E.; Di Renzo, F.; Quignard, F. Accessibility of the Functional Group of Chitosan Aerogel Probed by FT-IR-Monitored Deuteration. *Biomacromolecules* **2007**, *8* (11), 3646–3650.

(3) Nguyen-Thai, N. U.; Hong, S. C. Structural Evolution of Poly(Acrylonitrile-Co-Itaconic Acid) during Thermal Oxidative Stabilization for Carbon Materials. *Macromolecules* **2013**, *46* (15), 5882–5889.

(4) Sellitti, C.; Koenig, J. L.; Ishida, H. Surface Characterization of Graphitized Carbon Fibers by Attenuated Total Reflection Fourier Transform Infrared Spectroscopy. *Carbon* **1990**, *28* (1), 221–228.
